# Effectiveness of a one-year multi-component day-camp intervention for overweight children: study protocol of the Odense overweight intervention study (OOIS)

**DOI:** 10.1186/1471-2458-14-313

**Published:** 2014-04-05

**Authors:** Kristian Traberg Larsen, Tao Huang, Niels Christian Møller, Lars Bo Andersen, Mathias Ried-Larsen

**Affiliations:** 1 Centre of Research in Childhood Health, Institute of Sports Science and Clinical Biomechanics, University of Southern Denmark, Campusvej 55, Odense M DK-5230, Denmark; 2 The Centre of Inflammation and Metabolism (CIM) and The Centre for Physical Activity Research (CFAS), The Danish Diabetes Academy, Rigshospitalet, Section 7641, Blegdamsvej 9, Copenhagen DK-2100, Denmark

**Keywords:** RCT, Overweight, Children, Intervention, Physical activity, Weight loss, Camp intervention

## Abstract

**Background:**

Childhood overweight has noticeable psychological and social consequences for the child and leads to an increased risk of mortality and morbidity later in life. With the high prevalence of overweight in children and adolescents, it is important to identify effective approaches for the prevention and treatment of overweight in children and young individuals. The primary aim of the study is to assess the effect of an intensive day-camp intervention on body mass index (BMI) in overweight children.

**Methods:**

The Odense Overweight Intervention Study is a semi-blinded randomized controlled trial. Overweight children from the Municipality of Odense, Denmark, were invited to participate in the trial. Based on power calculations 98 participants were found to be sufficient to randomize in order to find an effect of minimum 1.5 BMI points. Gender-stratified concealed block randomization with a ratio of 1:1 and random block sizes of two, four, and six ensured balance between study arms. The intervention consisted of a six-week multi-component day camp including increased physical activity, healthy diet and health education followed by 46 weeks of family-based habitual intervention. The standard care arm was offered two weekly hours of physical activity training for six weeks. The outcomes were measured at baseline and at six-week and 52-week follow-ups. Furthermore, BMI will be assessed again at 48-month follow-up. Test personnel were kept blinded. The intervention effect will be evaluated using mixed model analyses. During 2012 and 2013, 115 children were enrolled in the study. Fifty-nine children were randomized to the day-camp intervention arm and 56 to the standard intervention arm.

**Discussion:**

This study will provide novel information about the long-term health effects of an intense day-camp intervention program on overweight children, due to the design and the follow-up period. Moreover, it will add to the knowledge on designing and implementing feasible camp settings for preventing overweight in children.

**Trial registration:**

NCT01574352 at http://clinicaltrials.gov on the 8th of March 2012.

## Background

Strong and consistent evidence has shown that overweight, including obesity, is a significant risk factor for the development of cardiovascular disease (CVD) in adults [1,2]. Several studies have found an association between overweight in childhood and increased risk of morbidity and mortality later in life [3-5]. Considering the high prevalence of overweight in children and adolescents, it is crucial to identify effective approaches for the prevention and treatment of overweight in children and young individuals [6,7]. According to a Cochrane review and meta-analysis from 2009, very few well-designed randomized controlled trials (RCT) have been conducted on the reversal of overweight in childhood and adolescence [8]. Of these studies, treatment strategies with the highest efficacy included combined diet, physical activity and other lifestyle interventions in both outpatient clinics and public school settings. Drug therapy showed an effect as well; however, these generally cause a range of adverse effects. Thus, drug therapy can be considered unsuitable to treat overweight in otherwise healthy children. When targeting prepubescent children, family involvement has been shown to be particularly effective [8]. Most studies conducted suffer from a lack of quality, such as low statistical power, high dropout, unadjusted outcome measures, and a relatively short follow-up time (<12 months). Consequently, well-designed high-quality studies are still needed on the reversal of overweight in childhood with increased focus on documenting predictors of behavior changes associated with clinical decreases in overweight. Approaches such as resident weight-loss camps have shown promising results [9-18]. A residential camp setting provides an opportunity to control exposure to, for instance, particular foodstuffs, beverages and physical activity opportunities. Some studies have shown a noticeable effect on body mass index (BMI) and other CVD risk factors after camp stays as short as two weeks. Generally, longer camp stays tend to be related to more pronounced effects [9,13,15]. However, little is yet known about the long-term effects of the residential camp approach. Gately et al. observed a decreased BMI in participants after an eight-week camp-based intervention program, both in the short term and at a ten-month follow-up [10]. Nonetheless, this study was uncontrolled and offered no intervention in the follow-up period. In a semi-controlled design, Nowicka et al. studied the effect of a 1-week resident sports camp with six months of supported follow-up intervention. They found no effect on overweight status after 12 months [16]. The short duration of the camp may explain the result. In an eight-week summer camp setting for overweight adolescents, Southam et al. invited parents to weekly meetings to discuss the program content, thereby involving parents more actively in changing the habits of their children. Despite significant effects observed at completion of the camp, no follow-up assessments were performed [13]. Thus, well-designed RCT with longer follow-up periods also involving the parents are needed to test whether an approach in which children are invited to stay at a camp setting, away from their everyday environment, can be effective in prevention of overweight. Earlier experiences with camps for overweight children in the municipality of Odense, Denmark, have shown promising results. Since 2005, the municipality has offered overweight fifth-grade children a six-week stay on a remote camp location, Fanoe Island, and a consecutive family-focused intervention (*Camp Fanoe*). Experiences from Camp Fanoe demonstrate that some children choose not to participate because of the distance and the six-week period away from their family [19]. Furthermore, the promising results potentially can be transferred to other settings. As a result the Odense Overweight Intervention Study (OOIS) was developed as a RCT, based on the framework and experiences from Camp Fanoe. However, the camp was moved to the center of the city of Odense (the third largest city in Denmark) and designed as a day camp instead of a residential camp.

### Study aims and hypothesis

The main purpose of the OOIS is to test the effect on BMI in a group of 11- to 13-year-old overweight and obese children participating in a one-year multi-component modified camp approach. The secondary aims of the study are to assess the intervention’s effect on CVD risk factors, cognitive skills, and motor skills. Furthermore, we aim to investigate the development of BMI four years subsequent to the initialization of the study. Our hypothesis is that the approach will induce a reduction of overweight in the intervention group compared to the control group over a one-year period. We aim to translate the research findings into intervention products and guidelines usable in practice in both national and international programs on prevention of overweight among children and youth. The primary aim of this paper is to describe the design and the study protocol of the OOIS.

## Methods

### Study design

The OOIS is a semi-blinded randomized controlled trial (RCT) with four measurement occasions. A population-based sample of overweight and obese children were invited to participate in either an already established resident overweight camp (Camp Fanoe) or the RCT. The participants choosing the RCT were randomly allocated to either the Day Camp Intervention Arm (DCIA) or the Standard Intervention Arm (SIA). The DCIA is a day camp lasting six weeks with a subsequent 46-week follow-up parent-supported intervention period. The SIA is a standard physical activity intervention consisting of one weekly activity class for six consecutive weeks.

### Recruitment and eligibility criteria

Approximately 4400 children from the municipality of Odense were invited to the mandatory annual schoolchild examinations in fifth grade from September to December 2011 and from September to December 2012. The children’s height, weight, and waist circumferences were measured by school health nurses. If participants exceeded age- and sex-specific BMI cut-points for overweight defined according to the International Obesity Task Force [20], they were informed about the two existing possibilities: the OOIS or Camp Fanoe. Criteria for exclusion from the OOIS were:

1. The child participated in other intervention research involving cardiovascular risk management.

2. The child did not attend a regular school or was attending a special class (“OBS” class), typically due to behavioral issues.

3. The child had a known endogenous cause of overweight.

4. The child had a motor-control handicap which hindered normal participation in physical activity.

5. The child had known violent behavior.

### Ethics and trial registration

Before initializing the study, the study protocol was approved by The Regional Scientific Ethical Committee for Southern Denmark (Approval number: S-20120015), registered with the Danish Data Protection Agency and at ClinicalTrial.gov (Registration number: NCT01574352). Written informed consent from children and their parents or legal guardians were obtained. Twice during the study the participants received parts of their personal results to keep them updated about their progression. The first of these was received after the first follow-up measurement and the second after the third follow-up.

### Sample size and randomization

**Figure 1:**
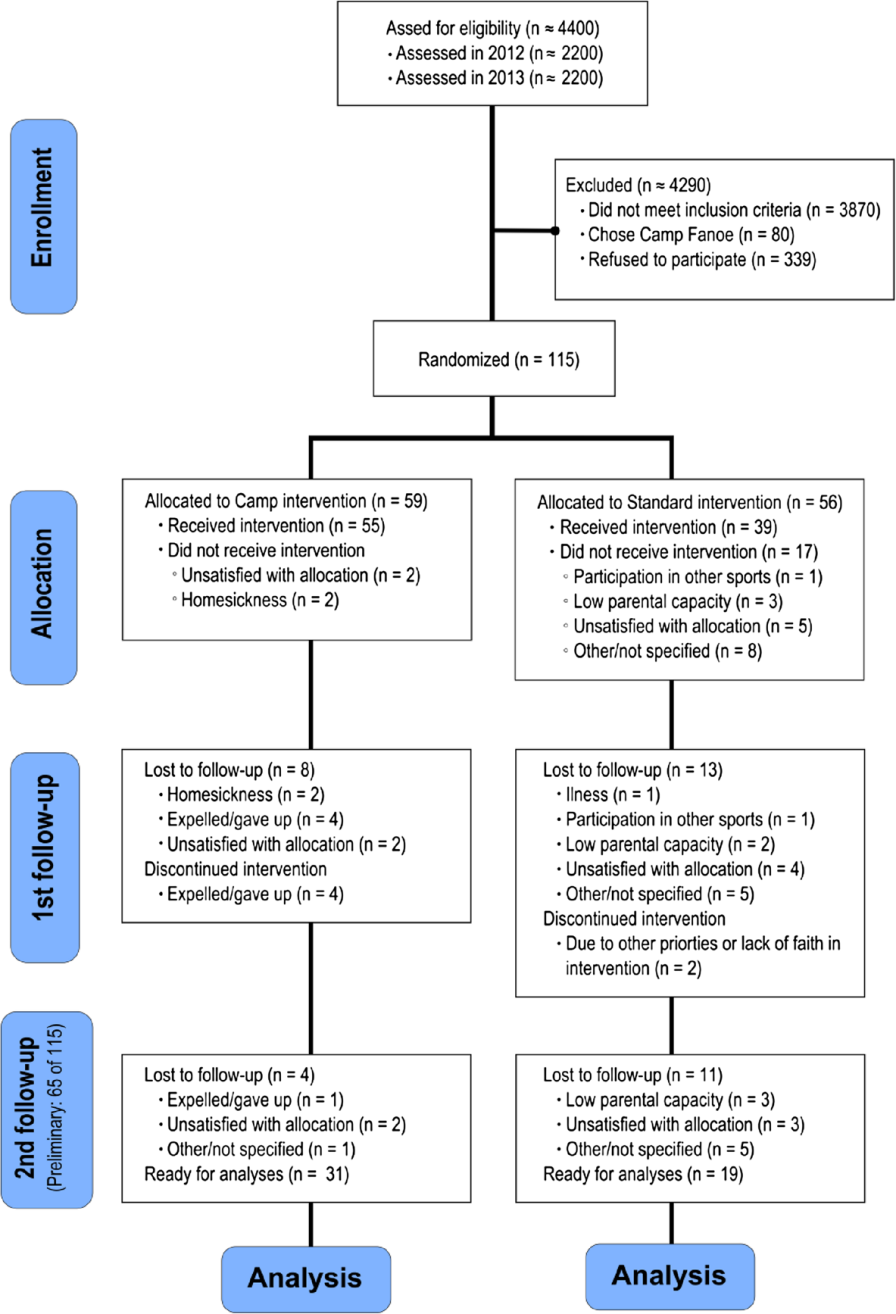
Diagram of preliminary participant flow.

It was expected that approximately 330 (15%) of a total of approximately 2200 fifth-grade children each school year would fulfill the eligibility criteria. Based on earlier experience we estimated that around 160 children would be interested in participating in the OOIS and/or Camp Fanoe. Each year Camp Fanoe enrolls 40 participants, which potentially leaves approximately 120 participants to choose the OOIS. During 2011–2012, 65 children were enrolled in the study, and during 2012–2013, an additional 50 participants were enrolled. Based on previous experiences from Camp Fanoe [19] and a pilot day camp conducted in 2011, a minimum difference of 1.5 BMI points (2.3 SD) between the DCIA and the SIA over 12 months was expected. Thus, 37 participants in each intervention arm were needed to achieve a power (1 - β) of 80% to find a significant difference between the intervention and control group (two-sided, α = 0.05) on the primary outcome (BMI). With an expected 30% attrition, 49 participants in each arm were to be recruited (total N = 98). To reduce attrition during the trial, children received a small gift (movie tickets) for participation at the three measurement occasions. Participation in either of the interventions was free of charge. Gender-stratified concealed block randomization with a ratio of 1:1 and a block size of two, four, and six (random permuted blocks) ensured balance between intervention arms. The randomization was generated using the web-based software http://www.randomization.com and http://www.random.org (flow of participants until spring 2013 is presented in Figure 1).

### Blinding and allocation concealment

Researchers were blinded at all assessments except for the ultrasonographer, due to his prior knowledge of the participants. The ultrasonographer was not involved in any other measurement procedures. Due to consideration for the participating families, and to avoid additional dropout on this account, it was necessary to inform participants of allocation three weeks prior to baseline measurements. Thus, allocation concealment was not possible.

### Theoretical approach

The theoretical framework of the intervention program is based on a psychosocial model of planned behavior change and the transtheoretical model (stages of change) at the individual level through systematic use of group processes [21]. The use of theory is supplemented by the practical experience obtained from Camp Fanoe.

### Day camp intervention arm

#### Family involvement

Family involvement had a central role in the DCIA on various levels. The families participated in an initial counseling session, with the aim of reaching family unanimity and securing the parents’ support of the children’s efforts to follow the designed program. Following this session, the children signed a *commitment contract* confirming their continuing involvement during the intervention. Parents received a handbook on dietary recommendations, a handbook concerning details in the camp program, and a pamphlet on child support. A parent council was founded before camp start to support parents in practical matters and augment participant involvement. Experiences from Camp Fanoe have revealed the positive influence of networking among parents and general parent involvement and support as a result of an active parent council. During the day camp the families were offered a dietary course led by a certified dietician. The course took place at the camp, where parents and children worked together to discuss healthy eating implementation in their families. The children were encouraged to commute actively if possible, preferably in smaller groups arranged according to residential area. Transportation was coordinated between the parents and the camp staff prior to the camp start.

### Content of the day-camp intervention arm

The day camps were located in the city of Odense, Denmark, and took place from mid-May to end of June in 2012 and 2013. The camp lasted for six consecutive weeks, seven days a week, and the children arrived every morning at 7 a.m. and left at 8.30 p.m. Except for commuting, the children typically stayed at home with their parents outside this time period. During a camp day the children were engaged in fun-based physical activity, sports, and health classes. Examples of activity classes include dancing, obstacle courses, teambuilding exercises, alternative ball games, adventure races, and more. All physical activity classes were planned and conducted by health professionals and specialist instructors and were primarily held in outdoor recreational facilities, such as soccer fields, basketball courts, and forest areas, within bicycling distance to the camp. Three hours of physical activity and sports were scheduled every day, and the classes were designed to reinforce motor skills, fitness, muscle strength, and confidence within the sporting environment. The planned exercise activities were both competitive and non-competitive, providing the children with both positive experiences in relation to physical activity and tools to cope with competitive situations in their normal environment. Every child was required to bring a bicycle to the camp. Besides scheduled physical activity the children were encouraged to stay physically active during breaks and leisure time. Six hours a week the children attended health classes with the purpose of increasing their knowledge on health issues such as the benefits of a healthy diet, sports participation, and increasing body awareness. The classes consisted of nutritional training, physiology, exercise, and goal setting. For this purpose appreciative inquiry (AI) was used to improve the potential for change within the children. This approach deals with inquiry into and dialogue about strengths, successes, values, hopes, and dreams related to lifestyle behaviors [22]. For at least one hour per day for five days a week, the children had time to focus on school work in order to follow the curricula of their classes in math, science, and Danish. The nutritional education consisted of a theoretical approach to the national Danish dietary recommendations in combination with personal guidance from the camp instructors during meals. At the camp, food was prepared and served according to the national Danish dietary recommendations [23]. The actual eating situation was supervised and guided by the camp instructors, but no calorie restriction was enforced.

### The subsequent family-based intervention

After the six-week intervention, a family-based (the participant and their parents/legal guardians) intervention consisting of four meetings during the subsequent 46 weeks was conducted targeting physical activity and dietary behavior. The meetings were led by trained school health nurses and instructors from the day-camp intervention. The following four themes were addressed during the four meetings: 1) healthy cooking and grocery shopping, 2) networking, 3) everyday physical activity, and 4) future challenges. The AI approach was applied in order to help the families handle the challenges related to behavioral changes. After the second meeting, an “activity and sports day” was arranged for the children by the camp instructors in order to give the children a supporting boost for the remaining intervention period. At all meetings, the families discussed and shared experiences related to the central topic of the meeting. The children had to be accompanied by at least one parent or legal guardian during these meetings.

### Standard intervention arm

As the Danish municipalities have no consensus in terms of how to offer help to obese children, no general standard intervention program can be used as comparison. Thus, it was decided to offer a shorter-term and less intensive intervention program compare to the day-camp group. This reflected a minimal effort to intervene in the children’s lifestyle and did not differ considerably from other initiatives being launched in Danish municipalities. This intervention consisted of one weekly fun-based physical activity session (1–2 hours duration) for six weeks, as well as a single health and lifestyle educational session for the parents, delivered by a dietician and physical activity specialist. The intervention program ended after six weeks.

### Measurements

A description of the measurements in the present study and time points of the assessments are presented in Table 1.

**Table 1:** Description of parameters, applied methods, and time points of measuring in the OOIS

**Parameter**	**Method of measurement**	**Measurement time points**			
		***Baseline***	***During camp***	***Follow-up 1 (post camp)***	***Follow-up 2 (post intervention)***
**Anthropometrics**					
BMI and body composition	Height assessed without footwear and weight in underwear on a Soehnle Professional Medical electronic scale. Waist circumference was assessed between the lower costal margin and the iliac crest to the nearest 0.5 cm, at the end of a gentle expiration. Hip circumference was assessed at the level of the great trochanter. Dual energy X-ray absorptiometry (DEXA) was performed by an experienced operator on a GE Lunar Prodigy (GE Medical Systems, Madison, WI), equipped with ENCORE software (version 12.3, Prodigy; Lunar Corp, Madison, WI).	√		√	√
Pubertal development	Pubertal development was assessed according to Tanner by self-evaluation to avoid violating the child’s privacy.	√		√	√
**Motor skills and fitness**					
Manual dexterity, aiming and catching, and balance	The objective part of the Movement-ABC II test battery was applied.	√		√	√
Hand grip strength	Hand grip strength was assessed using a Smedley Dynamometer (max. 100 kg).	√		√	√
Jump height	Jump height was assessed by measuring tape using the Sargent jump protocol. The best of three jumps was noted.	√		√	√
Cardiorespiratory fitness	Cardiorespiratory fitness was assessed using a progressive bicycle ergometer protocol (Monarch Ergomedic 839e) until total exhaustion with indirect calorimetry (Innovision, AMIS 2001) and a Polar RS800CX heart rate monitor.	√		√	√
Arterial health	Blood pressure was assessed on the left upper arm with an automatic blood pressure monitor (Welch Allyn 300 series) after 5 minutes of rest in a sitting position. Resting heart rate was assessed by heart rate monitor (Polar RS800CX) during the ultrasound assessment as the child already was in a horizontal position and relaxed. Intima-media thickness and carotid elasticity were assessed in a lateral and a posterior position on the carotid artery with a GE LOGIQ e portable ultrasound machine with an attached 12 L-RS probe.	√		√	√
**Blood measurements**	After an overnight fast, blood samples were drawn in the morning from the antecubital vein (right arm). Participants were lying supine during blood collection. Samples for serum were left at room temperature for 30 minutes. Samples for plasma (EDTA) were centrifuged as soon as possible and put in the icebox (within one hour) before centrifugation. All samples were centrifuged at 2500G for 10 minutes. Then the samples were stored at -80°C until analysis.				
CVD risk factors	Fasting blood samples were analyzed for insulin, glucose, HbA1c, and lipids.	√		√	√
Growth factors	Fasting blood samples were analyzed for BDNF and FGF-21.	√		√	√
Adipokines and myokines	Fasting blood samples were analyzed for leptin, adiponectin, and irisin.	√		√	√
Inflammatory factors	Fasting blood samples were analyzed for CRP, TNF-α, and IL-6.	√		√	√
**Cognitive function**	The testing environment was comfortable and free from distraction. The cognitive tasks were administered individually in the following order:				
Visual memory	Rey Complex Figure Test (RCFT)	√		√	√
Attention and processing speed	Symbol Digit Modalities Test (SDMT)	√		√	√
Attention and executive function	Trail Making Test A & B (TMT A & B)	√		√	√
Executive function	Stroop Color and Word Test	√		√	√
**Child and parent surveys**					
Child’s lifestyle, perceived built environment and well-being	By computerized questionnaire. Test personnel helped with the questionnaire if needed.	√		√	√
Parent’s lifestyle and socio-economic status	By letter-distributed questionnaire.	√			√
Parent’s evaluation of child’s executive function	By Behavior Rating Inventory of Executive Function (BRIEF).	√			√
**Habitual physical activity**	Assessed by hip-worn accelerometer Actigraph GT3X + for 10 consecutive days for habitual physical activity, for seven consecutive days for estimated activity level during the day camp (SCIA only), and for two occurrences of fun-based physical activity for the SIA.	√	√		√
**Food intake**	Assessed by food registration for two whole days at the camp. For every meal the child had their food weighed and photographed for subsequent analyses of energy and nutrition content by trained and qualified personnel.		√		

### Outcomes

The screening process for overweight was performed by school nurses by assessing height and weight, thus making it relevant to use BMI as the primary outcome. Moreover, BMI and BMI z-score are frequently used as primary outcomes in comparable overweight interventions. To complement and validate the BMI assessment, body composition by DEXA scanning will be introduced. Furthermore, as a secondary outcome, a modified composite metabolic risk z-score will be calculated based on the definition by Andersen et al. [24]. This modified version contains summed z-scores for systolic blood pressure, triglycerides, total cholesterol/HDL ratio, insulin resistance (HOMA-IR), abdominal fatness by waist circumference, and aerobic fitness.

### Additional data collection

The study planned to be object for research on the development of motor skills and cognition as well (and potentially more), and thus a broad range of assessments was performed. The children’s self-reported lifestyle and wellbeing were obtained by computerized surveys. The parents answered questionnaires on their socioeconomic status, lifestyle, and estimated executive function of their children. Most measurements were conducted on three occasions: at baseline (post-randomization), immediately after the day camp, and at 12-month post-baseline measurements. To estimate the habitual physical activity level of the children, accelerometer-based activity monitors were applied for a minimum of seven consecutive days before and after the one-year intervention period. During the day camp, the physical activity level was assessed by the monitors, and the energy intake was assessed by photographic recording combined with weighing during two complete days.

### Training of test personnel

The test personnel for the physical tests were employed students or Ph.D. fellows enrolled at the university. Prior to the baseline testing, the test team was trained by experienced testers within the relevant areas. To gain sufficient experience, the personnel practiced the tests repeatedly on a comparable target group of overweight children prior to the testing.

### Data treatment and validation

During physical tests, the data was noted on test sheets. Shortly after completion, the data was typed into Epidata software by two independent researchers. The same was done for parent questionnaires. The two versions were compared for misspellings and typos, and subsequently corrected if needed. Fitness data, heart rate data, accelerometer data, children’s questionnaire data, and DEXA scan data were extracted from appurtenant apparatus and transferred into the applied statistical software (STATA version 12) for further analyses.

### Analysis plan and statistics

Analyses and reporting of the results will follow guidelines from *the CONSORT statement*[25]. Descriptive statistics will be used to present participation and retention rates, potential year group differences (2012 vs. 2013), and group differences in age, BMI, body fat percent, fitness level, parents’ socioeconomic status, and ethnicity. Similar analyses will be performed to search for any potential cohort differences (2012 vs. 2013). To compare the effect of the intervention between arms, mixed models will be applied. The primary analyses will be done according to the *intention-to-treat* principle. Supplementary, a sensitivity analysis will be performed according to a *per-protocol* analysis in children adhering to the intervention protocol, and thus strict registration of participation will be conducted. Predefined decision rules specify that accepted adherence to the camp intervention requires an attendance of 85% of the time (corresponding to 34/40 days) for the DCIA and 4 out of 6 activity sessions for the SIA. The following sub-group analyses across baseline characteristics have been determined *a priori*: age, gender, SES of the parent (s), ethnicity, habitual physical activity, and motor skills. These sub-group analyses will be performed by testing for interaction between the abovementioned variables and the treatment arm. Other sub-group and effect-mediating analyses conducted *post hoc* will be considered exploratory.

### Timeframe and reporting

The OOIS recruited participants in the spring of 2012 and again in the spring of 2013 to reach sufficient participants to run the study. The duration of participation for each of the two year groups was approximately 52 weeks from baseline to second follow-up measurements. Thus, the last measurements will take place in the spring of 2014. The secondary part of the study is a four-year follow-up on the primary variable, and will take place in 2016 and 2017 in the school setting when the children are in the ninth grade, making the total duration of the study five years.

## Discussion

There has been a scientific and clinically broad interest in discovering feasible and effective methods to cope with the challenges of childhood overweight. Camps have been used as a setting for treatment of overweight for decades, but there is a lack of knowledge in terms of the actual effects. This study will provide novel information about the possible health effects of a day-camp intervention on overweight children, especially due to the RCT design and the 12- and 48-month follow-up period, respectively. Moreover, it will add to the knowledge on designing and implementing feasible camp settings for preventing overweight in children. A key strength of the study is the originality of the research. The literature indicates that so far no other studies of a camp-based intervention on overweight children with an RCT design and follow-up periods up to 12 and 48 months have been published. High-standard methods are applied for assessment of body composition, fitness, physical activity, and blood sample analysis. A voluntary sample of overweight children from the entire municipality further strengthens the study quality since a wide range of cultures and socioeconomic statuses will have the opportunity to participate. There are several limitations of the study. One worth mentioning is the lack of possibility to create a true control group in the study based on directions from the ethical committee. Both arms were required to receive an intervention, resulting in the standard intervention for the comparing group. As the power calculation was performed on the expected pronounced effect of BMI, the potential existing effects of secondary outcomes may not be revealed. To assess the intensity and amount of physical activity during the camp, accelerometers were applied for one week. However, this was only done during camp time, leaving it up to participants to self-report estimated additional physical activity, e.g. active/passive transportation. The children’s energy intake during the camp was estimated from two consecutive days of food registration during the camp, leaving the rest of the camp days unaccounted for. There was no recording of the food intake in the SIA. Furthermore, the children’s physical activity level during participation in the SIA was assessed by accelerometry solely on three of six occasions during their intervention period (1–2 hours each time).

## Competing interests

None of the authors have any competing interests regarding the OOIS or this manuscript describing the protocol for the study, and the study has not received any funding or assistance from a commercial organization.

## Authors’ contributions

MRL and LBA have been designing the original concept of camp Fanoe and the OOIS, and have been members of the steering committee during the planning and implementation of the study. KTL and TH have been planning and executed the practical part of the study, including planning the specifics of the interventions, recruiting participants, organizing tests of participants, and performing data collection. Moreover, KTL has drafted the study protocol and has collected and applied comments from co-authors. NCM has contributed scientifically to the protocol by advising on study design and manuscript development. All authors critically read and commented the manuscript and approved the final version.

## Pre-publication history

The pre-publication history for this paper can be accessed here:

http://www.biomedcentral.com/1471-2458/14/313/prepub
